# Author Correction: The impact of late-career job loss and genetic risk on body mass index: Evidence from variance polygenic scores

**DOI:** 10.1038/s41598-023-33157-4

**Published:** 2023-04-11

**Authors:** Lauren L. Schmitz, Julia Goodwin, Jiacheng Miao, Qiongshi Lu, Dalton Conley

**Affiliations:** 1 Robert M. La Follette School of Public Affairs, University of Wisconsin-Madison, 1225 Observatory Drive, Madison, WI 53706 USA; 2 Department of Sociology, University of Wisconsin-Madison, Madison, WI USA; 3 Department of Biostatistics and Medical Informatics, University of Wisconsin-Madison, Madison, WI USA; 4 Department of Statistics, University of Wisconsin-Madison, Madison, WI USA; 5 Department of Sociology, Princeton University & NBER, Princeton, NJ USA

Correction to: *Scientific Reports* 10.1038/s41598-021-86716-y, published online 07 April 2021

The Acknowledgements section in the original version of this Article was incomplete. It now reads:

“This research was funded by the following awards from the National Institute on Aging (NIA): K99 AG056599, R00 AG056599, P30 AG012846, T32 AG000221 (Schmitz), and P30 AG017266 (Lu and Schmitz). The content is solely the responsibility of the authors and does not necessarily represent the official views of the National Institutes of Health. The authors would like to thank Jason Fletcher, Rebecca Johnson, and Ramina Sotoudeh for helpful comments and feedback on earlier versions of this draft. This study deploys data from the Genetics Resource with the Health and Retirement Study (HRS), accession number phs000428.v2.p2.c1. These data were collected with financial support from the National Institute of Health’s (NIH) Director’s Opportunity for Research awards using American Reinvestment and Recovery Act funds (RC2 AG036495-01, RC4 AG039029-01). With these funds, the HRS has genotyped almost 20,000 respondents who provided DNA samples and signed consent forms in 2006–2012. The HRS data were produced and distributed by the University of Michigan under the directorship of David R. Weir, with funding from the National Institute on Aging (grant number NIA U01AG009470), Ann Arbor, MI. These data were accessed through dbGaP, under project number 14753 (P.I.: Dalton Conley, Princeton University).”

The original Article has been corrected.

