# Supplementary material for: The impact of late-career job loss and genetic risk on body mass index: Evidence from variance polygenic scores
Source: Sci Rep. 2021 Apr 7;11:7647. doi: 10.1038/s41598-021-86716-y (PMC8027610; doi:10.1038/s41598-021-86716-y)
Supplement: Supplementary file 1 — Supplementary Information. [file 41598_2021_86716_MOESM1_ESM.pdf]

Supplementary Information

The Impact of Late-Career Job Loss and Genetic Risk on Body Mass Index: Evidence from Variance Polygenic Scores

Lauren L. Schmitz[1], Julia Goodwin[2], Jiacheng Miao[3], Qiongshi Lu[3,4], Dalton Conley[5]

[1] Robert M. La Follette School of Public Affairs, University of Wisconsin-Madison

[2] Department of Sociology, University of Wisconsin-Madison

[3] Department of Biostatistics and Medical Informatics, University of Wisconsin-Madison

[4] Department of Statistics, University of Wisconsin-Madison

[5] Department of Sociology, Princeton University & NBER

Table S1. Variable definitions

Dependent variable	
BMI	Body mass index in kg/m^2
Independent variables	
Business closure	1=business closed; 0=still working for previous wave employer
BMI mPGS	Body mass index mean polygenic risk score, standardized
BMI vPGS	Body mass index variance polygenic risk score, standardized
Female gender	1=Female; 0=Male
Age	Age in years
Married	1=Married/partnered; 0=Divorced/separated/widowed/single
Highest degree obtained	Binary (0/1) variables for no degree or high school degree. The omitted category is associate's/bachelor's/professional degree.
Region dummies	Binary (0/1) variables for Census region of residence: Northeast (New England and Mid Atlantic); Midwest (EN Central and WN Central); South (S Atlantic, ES Central, and WS Central). The omitted category is West (Mountain and Pacific).
Household income (log)	Log of total (respondent + spouse) household income in 2010 dollars. Includes earnings, household capital income, income from all pensions and annuities, income from social security disability and supplemental social security income, income from social security retirement, spouse or widow benefits, income from unemployment or workers compensation, income from veteran's benefits, welfare and food stamps, alimony, other income, and lump sums from insurance, pension and inheritance.
Household wealth ($100k)	Total household income in 2010 dollars divided by 100,000 for scalar consistency. It is the sum of the value of primary residence, net value of real estate (not including primary residence), net value of vehicles, net value of businesses, net value of stocks, mutual funds, and investment trusts, value of checking, savings or money market accounts, value of CD, government savings bonds, and T-bills, net value of bonds and bond funds, and the net value of all other savings, less the value of all mortgages/land contracts (primary residence), value of other home loans (primary residence), and the value of any other debt.
Firm size[a]	Binary (0/1) variables for firm size categories: Less than or equal to 4 employees; 5-14 employees; 15-24 employees; 25-99 employees; 100-499 employees. The omitted category is firm size greater than or equal to 500 employees.
Part time	1=works part time; 0=does not work part time.
Industry[a]	Binary (0/1) variables for industry categories: agriculture, fishing, or farming; construction or mining; manufacturing; trade; public services; finance, insurance, or real estate; public administration. The omitted category is misc. services.

Occupational status[a]	Binary (0/1) variables for blue collar and service workers. The omitted category is white collar workers.
Job tenure[a]	Current job tenure in years.
Health status	1=excellent or very good self-reported health; 0=good, fair, or poor self-reported health.
Health insurance	1=covered by a federal or employer-sponsored health insurance program; 0=otherwise.
Exercise	1=exercises vigorously three or more times per week; 0=otherwise.
Ever smoke cigarettes	1=smoked 100 or more cigarettes in their lifetime; 0=otherwise.
Cigarettes per day[a]	Total number of cigarettes smoked per day, excluding pipes or cigars. Variable is set equal to zero if respondent does not smoke.
Drinks per week	Total number of alcoholic drinks per week
Doctor diagnosed psychiatric issue[a]	1=reports doctor diagnosed emotional or psychiatric problems; 0=otherwise.
Survey year	Binary (0/1) variables for 1994-2012. The omitted year is 1992.

[a]Variables with additional category for missing values. Analyses also controlled for the first 10 principal components of the European ancestry genetic data.

Table S2. Descriptive statistics of the sample (n=11,934)

Variable	Mean (SD) or n (%)	Min	Max
BMI	27.75 (5.25)	15.3	63.2
BMI mPGS	0.00 (1.00)	-3.061	3.229
BMI vPGS	0.00 (1.00)	-2.787	5.753
Female	57.16 (4.25)	0	1
Age	6,623 (4.25)	50	69
Married or partnered	9,675 (81.07)	0	1
No degree	724 (6.07)	0	1
High school degree	6,229 (52.2)	0	1
College degree	4,981 (41.74)	0	1
Household income (log)	11.33 (0.85)	0	15.763
Household wealth ($100k)	3.62 (13.72)	-10.079	1098.5
Works part time	1,373 (11.5)	0	1
Firmsize≤4	325 (2.72)	0	1
Firm size 5-14	693 (5.81)	0	1
Firm size 15-24	326 (2.73)	0	1
Firm size 25-99	1,252 (10.49)	0	1
Firm size 100-499	2,234 (18.72)	0	1
Firmsize≥500	6,889 (57.73)	0	1
Missing firm size info	215 (1.8)	0	1
Agriculture/Fishing/Farming	81 (0.68)	0	1
Construction/Mining	397 (3.33)	0	1
Manufacturing	1,967 (16.48)	0	1
Trade	1,498 (12.55)	0	1
Public Services	882 (7.39)	0	1
Finance/Insurance/Real Estate	761 (6.38)	0	1
Public Administration	776 (6.5)	0	1
Misc. Services	5,516 (46.22)	0	1
Missing industry info	56 (0.47)	0	1
White Collar	8,483 (71.08)	0	1
Blue Collar	2,154 (18.05)	0	1
Service	10,50 (8.8)	0	1
Missing occupation info	247 (2.07)	0	1
Job tenure	15.13 (10.86)	0	48.5
Missing tenure info	23 (0.19)	0	1
Health excellent/very good	7,610 (63.77)	0	1
Health insurance	9,684 (81.15)	0	1
Exercise vigorously 3+ times/week	4,322 (36.22)	0	1
Missing exercise info	41 (0.34)	0	1
Ever smoke cigarettes	6,414 (53.75)	0	1
Missing ever smoke cigarettes	54 (0.45)	0	1
Cigarettes per day	2.83 (7.78)	0	80
Missing cigarettes per day info	10,035 (84.09)	0	1

Drinks per week	2.35 (5.60)	0	84
Missing drinking	2,502 (20.97)	0	1
CES-D	0.76 (1.43)	0	8
Missing CES-D	1,586 (13.29)	0	1

Analytic sample consists of genotyped, European ancestry workers aged 50-70 who were not self-employed.

Table S3. Before treatment means of treated, controls, and matched controls

	Match status	Means		%bias	t-test p-value	V(T)/ V(C)
		Treated	Control			
BMI	U	27.35	27.76	-7.9	0.137	0.96
	M		27.53	-3.5	0.631	1.01
BMI mPGS	U	0.03	0.00	3.4	0.510	1.06
	M		0.05	-1.2	0.873	1.07
BMI vPGS	U	0.01	0.00	0.9	0.863	1.00
	M		0.04	-3.5	0.640	0.94
Female	U	0.54	0.56	-3.9	0.453	.
	M		0.54	-0.2	0.974	.
Age	U	57.43	57.15	6.3	0.212	1.15
	M		57.48	-1.2	0.869	1.15
Married	U	0.83	0.81	4.3	0.423	.
	M		0.82	2.6	0.715	.
No degree	U	0.11	0.06	18.2	<0.0001	.
	M		0.11	-0.2	0.981	.
High school degree	U	0.67	0.52	31.3	<0.0001	.
	M		0.61	12.3	0.088	.
College degree	U	0.22	0.42	-44.3	<0.0001	.
	M		0.28	-13.0	0.061	.
Household income (log)	U	11.08	11.34	-26.3	<0.0001	1.82
	M		11.17	-9.1	0.243	1.30
Household wealth ($100k)	U	3.10	3.64	-5.2	0.453	0.12
	M		3.33	-2.2	0.763	0.11
Works part time	U	0.19	0.11	21.5	<0.0001	.
	M		0.18	3.5	0.655	.
Firm size<=4	U	0.09	0.03	29.2	<0.0001	.
	M		0.08	7.0	0.423	.
Firm size 5-14	U	0.11	0.06	18.4	<0.0001	.
	M		0.09	6.3	0.429	.
Firm size 15-24	U	0.06	0.03	14.9	0.001	.
	M		0.04	7.2	0.367	.
Firm size 25-99	U	0.12	0.10	5.8	0.254	.
	M		0.11	3.4	0.646	.
Firm size 100-499	U	0.13	0.19	-17.6	0.002	.
	M		0.14	-4.2	0.542	.
Firm size>=500	U	0.38	0.58	-41.3	<0.0001	.
	M		0.42	-8.5	0.243	.
Agriculture/Fishing/Farming	U	0.02	0.01	11.0	0.004	.
	M		0.01	7.2	0.362	.
Construction/Mining	U	0.08	0.03	21.1	<0.0001	.
	M		0.06	7.7	0.351	.

Manufacturing	U	0.24	0.16	18.8	0.000	.
	M		0.20	8.2	0.279	.
Trade	U	0.26	0.12	36.2	<0.0001	.
	M		0.23	8.5	0.292	.
Public Services	U	0.07	0.07	0.3	0.954	.
	M		0.07	2.9	0.685	.
Finance/Insurance/Real Estate	U	0.06	0.06	0.1	0.985	.
	M		0.06	-0.2	0.984	.
Public Administration	U	0.01	0.07	-27.5	<0.0001	.
	M		0.04	-12.1	0.040	.
Misc. Services	U	0.25	0.47	-47.4	<0.0001	.
	M		0.32	-15.2	0.031	.
White Collar	U	0.64	0.71	-15.7	<0.0001	.
	M		0.65	-1.8	0.808	.
Blue Collar	U	0.24	0.18	15.1	0.002	.
	M		0.22	5.9	0.434	.
Service	U	0.11	0.09	8.3	0.095	.
	M		0.12	-4.0	0.613	.
Job tenure	U	11.36	15.25	-35.6	<0.0001	1.03
	M		12.54	-10.7	0.135	1.11
Health excellent/very good	U	0.62	0.64	-4.1	0.437	.
	M		0.61	0.8	0.914	.
Health insurance	U	0.65	0.82	-38.2	<0.0001	.
	M		0.69	-10.0	0.203	.
Exercise vigorously 3+ times/week	U	0.35	0.36	-1.6	0.759	0.99
	M		0.36	-1.3	0.859	0.99
Ever smoke cigarettes	U	0.62	0.53	17.6	0.001	.
	M		0.60	5.0	0.487	.
Cigarettes per day	U	5.30	2.75	27.4	<0.0001	1.94
	M		4.58	7.8	0.328	1.27
Drinks per week	U	2.24	2.35	-2.0	0.713	0.78
	M		2.10	2.7	0.709	0.78
CES-D	U	0.99	0.75	15.1	0.002	1.33
	M		0.95	2.5	0.747	1.00

Abbreviations: U, unmatched; M, matched; % bias: percent standardized bias; CES-D, Center for Epidemiological Studies-Depression 8 item scale. The t-test p-value refers to the p-value from the difference in means between the treated and control groups before and after matching. The V(T)/V(C) column presents the ratio of a continuous variable's variance for the treatment group over the variance for the control group. Additional covariates in the matching procedure: survey year, regional Census division, additional categories for variables with missing values, and the first 10 principal components of the European ancestry genetic data. We used kernel-based propensity score matching with a bandwidth of 0.06. Unmatched control observations=11,629; Unmatched treated observations=399; Matched control observations=11,559; Matched treated observations=375.

Table S4. Event time study analysis of differences in BMI between treatment and control groups pre-and post-job loss

	(1)	(2)	(3)
	Full analytic sample	High vPGS	Low vPGS
	Beta (SE)<br>95% CI<br>*p*-value	Beta (SE)<br>95% CI<br>*p*-value	Beta (SE)<br>95% CI<br>*p*-value
t-6	-0.198 (0.148)	-0.382 (0.240)	0.0106 (0.185)
	[-0.49, 0.09]	[-0.852, 0.089]	[-0.351, 0.373]
	0.182	0.112	0.954
t-4	-0.063 (0.127)	-0.274 (0.185)	0.160 (0.179)
	[-0.312, 0.186]	[-0.637, 0.090]	[-0.190, 0.511]
	0.62	0.14	0.37
t-2 (reference category)	-	-	-
t	-0.109 (0.109)	0.041 (0.144)	-0.313 (0.155)
	[-0.323, 0.104]	[-0.242, 0.323]	[-0.616, -0.009]
	0.315	0.776	0.043
t+2	0.047 (0.157)	-0.034 (0.205)	0.079 (0.242)
	[-0.260, 0.354]	[-0.436, 0.369]	[-0.396, 0.553]
	0.763	0.87	0.745
R-squared	0.884	0.887	0.888
Observations	12,700	6,348	6,352
Treated observations	1,192	619	573
Control observations	11,508	5,729	5,779

Abbreviations: SE, standard error; CI, confidence interval. Robust standard errors in parentheses. Regressions were run separately for the full analytic sample (Column 1), and then stratified by high versus low vPGS groups (Columns 2 and 3). vPGS groups were stratified at the median vPGS value. All specifications adjust for BMI in the previous wave, or BMI(t-2), and for the conditioning variables used in the propensity score matching that are reported in Table 1 and defined in detail in Supplementary Table S1. Individuals in the treated and control groups can have multiple observations. Unique N(treated)=428; unique N(control)= 3,562.

Table S5. Mean within-person BMI by vPGS quartile

	BMI					Number of waves BMI observed			
Quintiles of vPGS	Mean	SD	Min	Max	Obs.	Mean	SD	Min	Max
Quartile 1 (lowest)	27.73	5.14	17.2	54.4	3,603	6.924	3.33	2	13
Quartile 2	28.03	5.31	16.1	55.8	3,605	7.051	3.35	2	13
Quartile 3	27.76	5.35	15.9	63.2	3,603	7.146	3.37	2	13
Quartile 4 (highest)	28.12	5.52	16.0	55.8	3,601	6.918	3.26	2	13

Abbreviations: SD, standard deviation; Obs., observations. BMI statistics were calculated for control and treated individuals for all waves they were observed in the HRS between the ages of 50 and 70 (regardless of whether or not all waves were included as treated or control observations in the final analytic sample). Person-wave observations (N=14,412). Total unique observations (N=3,939).

Table S5 (Continued). Within-person BMI by quartile of vPGS and treatment status using panel sample

	Treated					Control				
Quintiles of vPGS	Mean	SD	Min	Max	Obs.	Mean	SD	Min	Max	Obs.
Quartile 1 (lowest)	28.00	5.14	17.2	47.5	715	27.71	5.16	17.2	54.4	2,890
Quartile 2	27.93	5.63	17.1	55.8	712	27.98	5.19	16.1	54.9	2,891
Quartile 3	27.31	5.37	15.9	47.5	719	27.87	5.32	15.9	63.2	2,891
Quartile 4 (highest)	27.76	5.21	16.0	50.2	707	28.25	5.62	16.6	55.8	2,887

See legend above. Person-wave observations (N=14,412). Total unique observations (N=3,939).
